# Colonic intussusception in descending colon: An unusual presentation of colon lipoma

**Published:** 2016-12

**Authors:** Reza Bagherzadeh Saba, Amir Sadeghi, Neda Rad, Mohammad Taghi Safari, Farnoush Barzegar

**Affiliations:** 1 *Gastroenterology and Liver Diseases Research Center, Research Institute for Gastroenterology and Liver Diseases, Shahid Beheshti University of Medical Sciences, Tehran, Iran*; 2 *Basic and Molecular Epidemiology of Gastrointestinal Disorders Research Center, Research institute for Gastroenterologyand Liver Diseases, Shahid Beheshti University of Medical Sciences, Tehran, Iran*

**Keywords:** Colonic lipoma, Colonic intussusception, Descending colon

## Abstract

Lipomas of the colon are relatively rare benign soft tissue tumors derived from mature adipocytes of mesenchymatic origin. During colonoscopy, surgery or autopsy they are generally discovered incidentally. Most cases are asymptomatic, with a small tumor size, and do not need any special treatment. However, in the cases with larger in size of tumor some symptoms such as anemia, abdominal pain, constipation, diarrhea, bleeding, or intussusception may be presented. We reported a 47-year-old woman with colonic intussusception in the descending colon caused by colonic lipoma and diagnosed after surgical exploration for obstructive colonic mass.

## Introduction

Colonic lipoma is rare submucosal benign tumor of the gastrointestinal tract. It is the third most common benign tumor with the incidence ranging between 0.2% and 4.4%, following hyperplastic and adenomatous polyps (1, 2).As they are usually asymptomatic, most cases are found incidentally during colonoscopy, surgery or autopsy (3, 4).However, in 30% of cases with larger size of tumor different symptoms such as anemia, abdominal pain, constipation, diarrhea, bleeding, or intussusception may developed (5). Despite the fact that imaging methods and endoscopic interventions has been improved recently, ultimate diagnosis is still hard. In cases with large colon lipomas, differentiation from malignancy is sometimes impossible before surgery and accurate diagnosis will be made after histopathological evaluation (6).In this study, we present a rare case of giant descending colonic lipoma causing a colo-colonic intussusception.

## Case report

**Figure 1:**
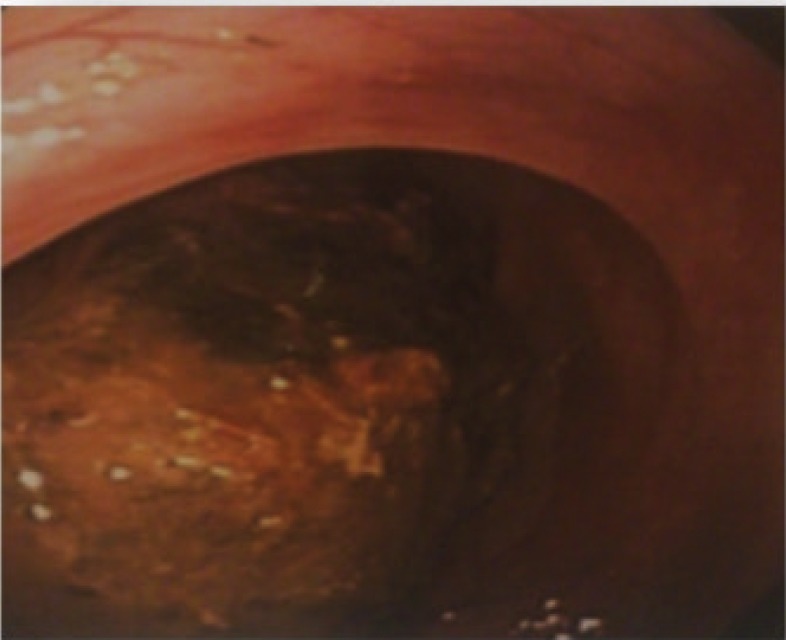
Colonoscopic view of polypoid lesion protruding into the colonic lumen

**Figure 2:**
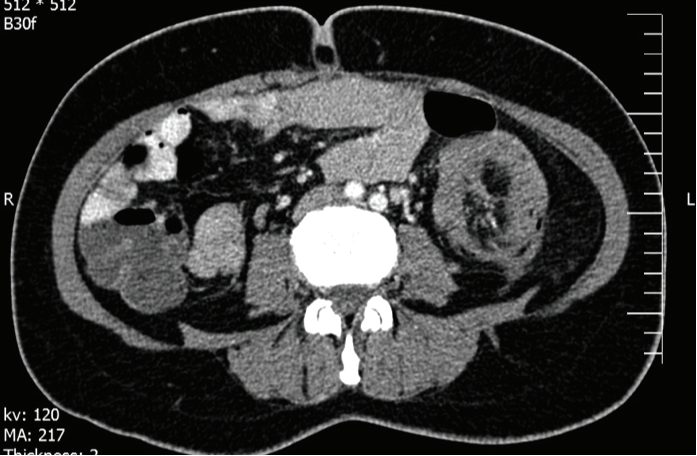
Tomographic view of the intussuscepted descending colon

**Figure 3:**
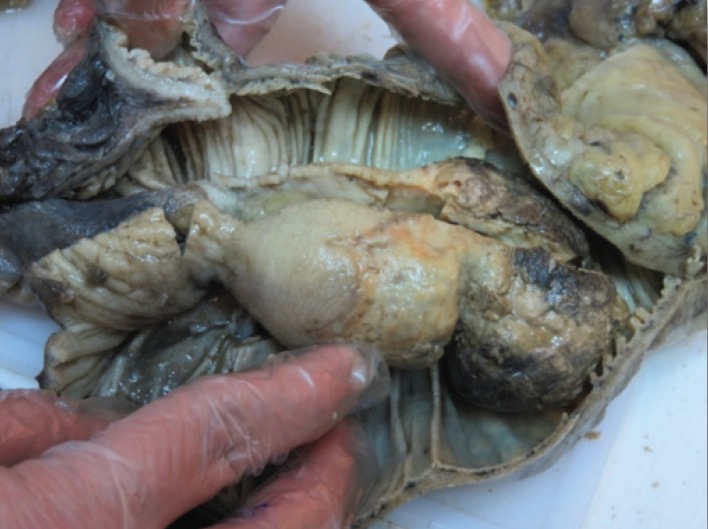
Gross morphology of lesion

**Figure 4:**
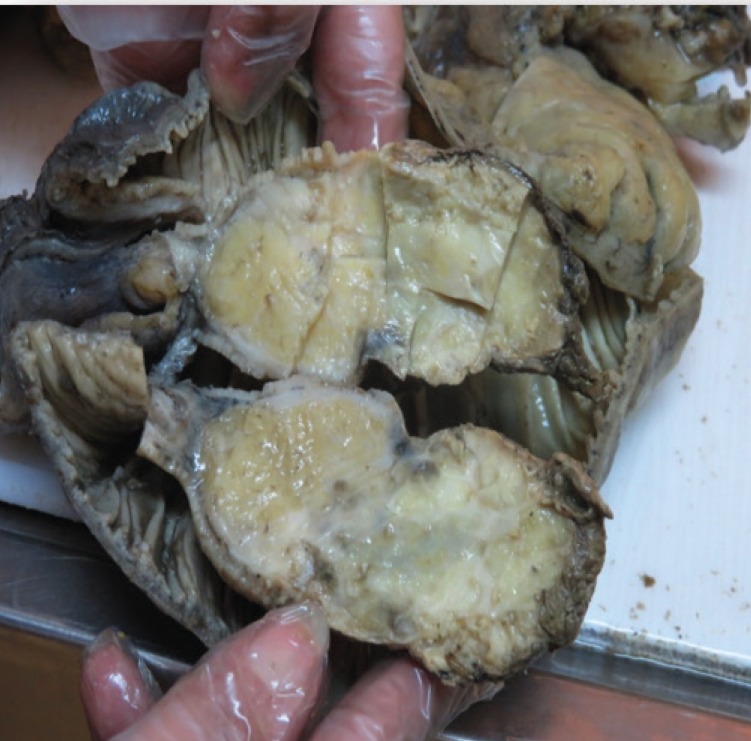
Resected specimen representing fatty consistency

A47-year-old woman was admitted to our hospital with four years history of constipation requiring laxative consumption. Her symptoms became severe in the last two weeks. Physical examination revealed abdominal distension and hyperactive bowel sounds. Laboratory results were within the normal range. Colonoscopy showed a large necrotic mass in proximal portion of descending colon, near splenic flexure with severe narrowing of lumen and bezoar like feces (figure 1). Histopathologic examination of the specimens revealed just some mucous material and autolyzed tissue,and definite diagnosis could not be made. Computed tomography was done and showed colo- colonic intussusception in the level of descending colon and sigmoid secondary to large fat density intramural mass as a lead point. Mild dilation of the ascending and transverse colon were seen (figure 2). The patient underwent a laparoscopic surgery, which confirmed the diagnosis of colo-colonic intussusception and then laparoscopic resection of descending colon and primary anastomosis was done. A large (13cm in length and 5cm in diameter) pedunculated polypoid mass was found as a lead point (figure 3 and 4). Histological examination confirmed lipoma. Three excised lymph nodes were reactive. Three days after surgery, the patient was discharged and she was symptomless.

## Discussion

Colonic lipomas are infrequent benign adipose tumors with an incidence ranging from 0.2% to 4.4% around the world.

They are mainly found in women between 50 and 60 years old (4, 7-9). Generally, colonic lipoma presents as sessile polypoid structure, originating from the submucosa with an intact mucosa. Commonest location for this benign lesion is on the right side, particularly in the cecum (10).Precisely, Lipoma of the colon is predominantly localized in ascending colon (61% from the reported cases), follow by descending colon (20.1%), transverse colon (15.5%) and in the rectum (3.4%). Multiple lipomas have been shown in 10-20% of the patients, notably if a lipoma was found in the cecum (11). Colonic lipomas are usually asymptomatic but a few of the lipomas have clinical manifestations and symptoms correlated to the size and location of the tumor. Signs and symptoms are more generally related with lipomas larger than2 cm and including abdominal pain, constipation, rectal hemorrhage and rarely with intussusception, as in our case.

Adult intussusception is rare clinical entities which constitute only 1–5% of all cases presenting as bowel obstruction. Lipomas are the most frequently occurring benign tumors of the colon which cause adult colonic intussusception. Contrary to other reported in the literatures (12, 13), surgical finding in our patient was intussusception with pedunculated lipoma as lead point in descending colon.

The preoperative diagnosis is usually very difficult but is important for planning. There are different imaging modalities for the preoperative diagnosis of colonic lipomas. Barium enema is not specific and the lesion can be mistaken for any other type of colonic neoplasms. Computed tomography (CT) scan is considered to be the study of choice for colonic lipomas because the mass typically presents a characteristic fatty densitometric value. This test visualizes spherical or ovoid mass and homogenous internal lesions with an absorption density of -40 to -120 Hounsfield units. CT scan is particularly useful in the detection of lipomas larger than 2 cm.

However, for small lipomas, the diagnostic value of CT is low. In fact, the diagnostic value is limited by the size and partial volume of the lesion.

Some investigators have recommended enteroclysis for intussusception diagnosis, but only one case has been reported (12). Magnetic resonance imaging has been recently used successfully, but further evaluation is still necessary. Colonoscopy is a modality which allows direct visualization of lipoma; a benign tumor present as a yellow, smooth mass with pedunculated or sessile base. Endoscopic findings that are characteristic of lipoma ‘cushion sign’ (forcing the forceps against the lesion results in depression and then restoration of the mass) and ‘naked fat sign’ (fat extrusion during the biopsy).A barium enema series was not performed in our patient. Instead, colonoscopy and abdominal CT scans were used for diagnosis. We performed preoperative colonoscopy in our patient to help for diagnosis of the organic lesion but colonoscopy showed only large necrotic mass in proximal of descending colon, near splenic flexure with severe narrowing of the lumen and bezoar like faces. Computed tomography of the abdomen in our case revealed colo-colic intussusception with the lead point being a lipoma of descending colon wall. Overall, CT is an excellent method to diagnose giant colonic lipomas.

In view of the uncertain etiology and diagnosis and high incidence of malignancy (approaching 50%), the treatment of intussusception in adults is invariably surgical resection (2, 4-6). The majority of authors recommend surgery as the standard method of treatment for every colonic lipoma greater than 2 cm in size (4,6). Surgical treatment includes resection, colotomy with local excision, limited colon resection, segmental resection, hemicolectomy, or subtotal colectomy. The choice of any of the above mentioned surgical interventions mainly depends on the lipoma size, location, and the presence or absence of definite preoperative diagnosis or disease complications. The time and the type of the surgical intervention differ and depend on the site, cause, and degree of obstruction. Most surgeons agree that resection is necessary, particularly in colonic intussusceptions and in older patients, because of the possibility of a malignant tumor. Some authors have recommended a selective approach to resection, depending on the site of intussusception, which influences the type of pathology (1, 14). Chang and colleagues (15) recommended operative reduction for small-bowel intussusceptions but not for colonic intussusceptions. Gupta and colleagues (16) reported resection in 70% of colonic intussusceptions. During last years a few selected cases of successful laparoscopic resection under colonoscopic guidance of symptomatic colonic lipomas have been reported (17,18). Endoscopic mucosal resection using the electrocautery snareis preferred technique for the excision of lipomas smaller than 2 cm in size. However, endoscopic removal of sessile or broadly based lipomas may result in a high rate of perforation and hemorrhage. Most authors believe that surgical resection is the ideal choice of treatment for every colonic lipoma greater than 2 cm in size. Colotomy excision or segmental colon resection is indicated when the diagnosis is definite. On the other hand, a segmental resection, hemicolectomy or subtotal colectomy is recommended when diagnosis is questionable or when a complication occurs. It is recommended to use laparoscopic resection in the face of a known lipoma because the patient enjoys the benefit of shorter period of ileus, shorted disability, less postoperative pain, and shorter hospital stay and recovery period (14). In our case laparoscopic surgery was performed successfully. Easily diagnosis of colonic lipoma, colonoscopy with biopsy is generally recommended. A general procedure can help discern a benign colonic lipoma from other diseases is abdominal CT scan (15). Management of the colonic lipoma depends upon their clinical picture, size and location is involves in endoscopy and laparoscopy removal and traditional open surgery(16). Considering that lipomas are most often located in the ascending colon, we present a rare case of a giant colonic lipoma of the left colon. Indeed our specific case of an incidentally discovered of a colonic lipoma that meets the typical gender, and symptoms of this tumor but not the typical size, location and age; the patient had a large mass (13*5 cm) with an atypical site (descending colon).
